# Genetic basis of maize maternal haploid induction beyond *MATRILINEAL* and *ZmDMP*

**DOI:** 10.3389/fpls.2023.1218042

**Published:** 2023-10-04

**Authors:** Henrique Uliana Trentin, Matheus Dalsente Krause, Rajkumar Uttamrao Zunjare, Vinícius Costa Almeida, Edicarlos Peterlini, Valeriy Rotarenco, Ursula Karoline Frei, William Dale Beavis, Thomas Lübberstedt

**Affiliations:** ^1^ Bayer Crop Science, Coxilha, RS, Brazil; ^2^ Department of Agronomy, Iowa State University, Ames, IA, United States; ^3^ Division of Genetics, ICAR-Indian Agricultural Research Institute, New Delhi, India; ^4^ Federal University of Viçosa, Viçosa, MG, Brazil; ^5^ Department of Agronomy, State University of Maringá, Maringá, PR, Brazil; ^6^ VAR BREEDING Ltd., Chisinau, Moldova

**Keywords:** maize maternal haploid induction rate, maize haploid inducers, genome-wide association study, maize doubled haploid technique, single fertilization

## Abstract

In maize, doubled haploid (DH) lines are created *in vivo* through crosses with maternal haploid inducers. Their induction ability, usually expressed as haploid induction rate (HIR), is known to be under polygenic control. Although two major genes (*MTL* and *ZmDMP*) affecting this trait were recently described, many others remain unknown. To identify them, we designed and performed a SNP based (~9007) genome-wide association study using a large and diverse panel of 159 maternal haploid inducers. Our analyses identified a major gene near *MTL*, which is present in all inducers and necessary to disrupt haploid induction. We also found a significant quantitative trait loci (QTL) on chromosome 10 using a case-control mapping approach, in which 793 noninducers were used as controls. This QTL harbors a kokopelli ortholog, whose role in maternal haploid induction was recently described in Arabidopsis. QTL with smaller effects were identified on six of the ten maize chromosomes, confirming the polygenic nature of this trait. These QTL could be incorporated into inducer breeding programs through marker-assisted selection approaches. Further improving HIR is important to reduce the cost of DH line production.

## Introduction

Today’s maize maternal haploid inducers have substantially higher induction rates than paternal haploid inducers, and are, therefore, the preferred tool for DH line production (Gilles et al., 2017a). The induction ability of an inducer is estimated by the rate of seeds with haploid embryo over the total number of seeds produced in cross-pollinations with a donor genotype. This rate is referred to as haploid induction rate (HIR) and is usually expressed as a percentage. In paternal inducers, where HIRs of up to 6% were observed (Kindiger and Hamann, 1993), haploid induction is attributed to a single gene, called *indeterminate gametophyte1* (*ig1*) (Kermicle, 1969). In maternal inducers, where breeding efforts raised HIRs from 3.2% to 14.5% (Coe, 1959; Rotarenco et al., 2010), HIR is under polygenic control (Lashermes and Beckert, 1988; Deimling et al., 1997; Röber, 1999; Prigge et al., 2012a; Prigge et al., 2012b; Liu et al., 2015). The first study designed to investigate the genetic nature of maternal haploid induction was performed by Lashermes and Beckert (1988), who observed variation in the induction ability of different segregating populations derived from crosses between inducer Stock 6 (Coe, 1959) and various noninducer lines.

Two quantitative trait loci (QTL), located on chromosomes 1 and 2, were identified in a F_3_ population derived from Stock 6, jointly explaining 17.9% of the phenotypic variance (Deimling et al., 1997). Studies conducted by Röber (1999) and Barret et al. (2008) confirmed the presence of a major QTL on chromosome 1 and reported strong segregation distortion against the inducer allele at this locus. A QTL mapping study conducted by Prigge et al. (2012b), which used multiple filial generations derived from four different bi-parental crosses, located this major QTL (*qhir1*) to bin 1.04 and found additional QTL on chromosomes 3 (*qhir2* and *qhir3*), 4 (*qhir4*), 5 (*qhir5* and *qhir6*), 7 (*qhir7*) and 9 (*qhir8*). *qhir8* explained more than 20% of the genetic variance in all filial generations of the cross between inducers CAUHOI and UH400 (Prigge et al., 2012b). Dong et al. (2013) subsequently fine mapped *qhir1* to a 243 kb region and observed large variation in HIR among F_2:3_ progeny homozygous for this locus. Their findings are in agreement with observations made by Prigge et al. (2012b), who noted that while *qhir1* is required for the formation of seeds with haploid embryos, multiple segregating alleles affect HIR. A conditional haplotype extension (CHE) test designed to detect selective sweeps under the assumption of confounding trait expression and population structure, identified two distinct loci (*qhir11* and *qhir12*) within *qhir1* (Hu et al., 2016). Subsequently, Nair et al. (2017) demonstrated the importance of *qhir11* only in haploid induction by genetically delineating the *qhir1* locus.

Subsequently independent researchers discovered that a frameshift mutation in a gene underlying *qhir11*, and coding for a patatin-like phospholipase, triggers the formation of haploid seeds (Gilles et al., 2017b; Kelliher et al., 2017; Liu et al., 2017). This frameshift mutation is caused by a 4-bp insertion in the last exon of gene Zm00001d029412, which leads to an early stop codon and results in a truncated protein, losing its proper localization at the pollen endo-plasma membrane (Gilles et al., 2021). The almost simultaneous discovery of the function of this gene by different researchers resulted in different names: *MATRILINEAL (MTL)* (Kelliher et al., 2017), *Zea mays Phospholipase A1 (ZmPLA1)* (Liu et al., 2017) and *NOT LIKE DAD (NLD)* (Gilles et al., 2017b). By inducing mutations close to the 4-bp insertion site in a noninducer, Kelliher et al. (2017) observed HIRs ranging from 4.0% to 12.5% in T1 and derived plants. This suggest that mutations in *MTL* are capable to generate inducers with high HIRs. However, the same 4-bp insertion was found in all inducers sequenced by Kelliher et al. (2017); Liu et al. (2017) and Gilles et al. (2017b), which have HIRs ranging from 2.0% to 10.0%. This is consistent with the presumed polygenic control of HIR (Lashermes and Beckert, 1988; Deimling et al., 1997; Röber, 1999; Prigge et al., 2012b; Liu et al., 2015). Zhong et al. (2019) identified Zm00001d044822, which encodes for a DUF679 domain membrane protein, to be the underlying cause of *qhir8*. The authors named this gene *ZmDMP* and confirmed its ability to enhance the HIR of inducers possessing *mtl/zmpla1/nld*, as previously reported by Liu et al. (2015). They also observed that in genotypes homozygous for the wild type *MTL/ZmPLA1/NLD* allele, knockout mutations in *ZmDMP* can disrupt haploid induction at very low frequencies (0.1-0.3%). This was an interesting observation because it indicated that mutations in different genes can promote and/or have synergetic effect on maternal haploid induction (Jacquier et al., 2020 and Jacquier et al., 2021).

The savings from employing inducers with higher HIRs can be substantial and are important to breeding programs where haploid selection is visually performed through the *R1-nj* color marker (Chase and Nanda, 1965). This dominant allele leads to anthocyanin production in the scutellum and aleurone layer of seeds in which double fertilization and embryonic development occurred normally. However, since embryos of haploid seeds do not contain inducer chromosomes, they also lack anthocyanin pigmentation. This difference in embryo pigmentation allows differentiation of haploid and diploid seeds. The savings from employing more efficient inducers can be significant, since the number of donor seeds that will be planted, and whose plants would be detasseled, harvested, dried, and screened for haploid seeds directly depends on the inducers’ HIR and the number of DH lines that one wants to extract from that donor population. To gain better understanding of the genetic basis of HIR, we designed a genome-wide association study (GWAS) using a diverse set of haploid inducers adapted to Midwestern U.S. conditions. The objectives of this study were to (i) identify promising maternal inducers to be used as parents of new inducer breeding populations, (ii) locate and assess the effect of QTL affecting HIR, and (iii) gain better understanding of the biological processes underlying haploid induction by identifying candidate genes within the detected QTL.

## Materials and methods

### Germplasm

We used a diverse panel of North American and European haploid inducers to identify QTL affecting HIR. The North American panel contains inducers in the background of public and Ex-PVP lines, such as A637, B73, DK78004, LH82, Mo17, PHG50 and Va35 (**Table S1**). All lines from the North American panel have the cross between RWS (Röber et al., 2005) and RWK (Röber et al., 2005) as their source of *mtl/zmpla1/nld*. The European panel contains lines such as MHI (Chalyk, 1999), PHI-3 (Rotarenco et al., 2010), RWS, RWK and eight F_7_ progeny derived from the cross of RWS and PHI-3, which were selected for high HIR. In total, 159 inducers were evaluated for HIR. The HIR of all inducers was evaluated in crosses with the commercial hybrid Viking 60-01N, from Albert Lea Seed Company (Minnesota, US). This hybrid was chosen as a donor because it possesses good inducibility and allows clear expression of the *R1-nj* marker. Inducibility is the ability of the donor parent to generate haploid seeds, and multiple studies indicated that the source germplasm impacts HIR (Randolph, 1940; Chase, 1952; Lashermes and Beckert, 1988; Eder and Chalyk, 2002; De La Fuente et al., 2018; Nair et al., 2020; Ren et al., 2022; Trentin et al., 2022).

A panel of 793 non-inducer genotypes was considered for a case-control GWAS (cc-GWAS) (**Table S1**). This panel was composed mainly of DH or highly homozygous lines derived from various genetic backgrounds, such as B73, BS39 (Verzegnazzi et al., 2021; Santos et al., 2022), several cycles of the Iowa Stiff Stalk Synthetic Population (BSSS) (Ledesma et al*.*, in preparation), BGEM (Iowa State University/USDA-ARS Germplasm Enhancement Maize project) and Ex-PVP lines. No haploid plants were observed when these genotypes were grown in the field, which is in accordance with Chase (1947) observation that the occurrence of haploid plants in elite inbred lines happens at very low frequencies (<0.1%).

### Field plot design

The inducers and donor used for this experiment were sown at the Iowa State University, Agronomy and Agricultural Engineering Farm, located in Boone (Iowa), during the summer of 2018. The trial was grown under rainfed conditions, following the recommended practices for maize production in Central Iowa. Pre- and post-emergence herbicides along with manual weeding were used to control invasive species. Urea ammonium nitrate was applied in the area before sowing. Two blocks of inducers and donor were sown at different planting dates to ensure that enough seeds would be generated to obtain reliable estimates of HIR. The first block of donor seeds was sown on May 8^th^, and the first block of inducers seeds was sown on May 21^st^. The second block of donor seeds was sown along with the first block of inducers seeds, while the second block of inducers seeds was sown on May 31^st^. Sowing of inducer blocks was delayed because most inducers have a significantly earlier maturity than the donor.

With the delayed planting of the haploid inducers, it was possible to pollinate the first donor block exclusively with the first inducer block, and the second donor block exclusively with the second inducer block. Each inducer and donor block was composed of subblocks containing 16 plots. Inducers were not randomized among subblocks because the great difference in vigor among them would adversely affect other traits for which data were collected for a companion study (Trentin et al., 2023). This rationale also justified sowing closely related inducers side-by-side within each subblock. For instance, if hybrid and inbred inducers were randomized, the shading caused by differences in plant height would be detrimental to the growth and development of inbred inducers. Plots were 5.5 meters long, 0.75 meters wide, and were sown with 25 seeds. Inducer and donor blocks were sown side-by-side, and pollen from inducers in a given subblock was carried to the adjacent donor subblock. Multicolored tags with easy-to-match codes were used to ensure that pollen from each inducer plot was placed in the corresponding donor plot. Bulk pollen was collected in tassel bags and used to pollinate at least 10 ears of the donor, which were covered before silk emergence using wax bags. Ears were harvested when seeds reached the black layer stage and were air-dried for one week.

### Data collection

Visual haploid selection was performed using the *R1-nj* marker, and the number of putative haploid and diploid seeds of each ear was recorded. Embryo and endosperm abortion, which are correlated with HIR (Prigge et al., 2012b; Xu et al., 2013; Nair et al., 2017), occur at different stages of seed development (Xu et al., 2013). This makes the identification of embryo and endosperm aborted seeds quite subjective, and for this reason, these two classes of seeds were not considered as a category in the statistical analysis of the data.

### Ploidy determination of the putative haploid seeds

Putative haploid seeds produced by each inducer at each planting date were bulked in a single envelope, whereas putative diploid seeds were discarded. The ploidy of the putative haploid seeds was verified by cutting seeds in half and observing the presence of anthocyanin pigmentation in the embryonic region. With the true number of haploid seeds, a weighted correction factor $\left(CF_{i}\right)$ for the $i^{th}\;$ inducer was calculated as follows:


$$CF_{i}=\frac{n_{1}\frac{TH_{1}}{PH_{1}}\;+\;n_{2}\frac{TH_{2}}{PH_{2}}}{n_{1}+\;n_{2}}$$

Where the indices 1 and 2 stand for the first and second planting dates, respectively, TH is the true number of haploid seeds, PH is the putative number of haploid seeds, and n is the number of harvested ears. The $CF_{i}$ was then used to correct the number of putative haploid seeds for the $i^{th}\;$ inducer in each planting date, where the difference between the counts before and after the correction was re-classified as diploid seeds.

### Statistical analysis of phenotypic data

Two modeling strategies were implemented to analyze and understand the variability of the phenotypic data by considering planting dates as blocks (Silva, 2017; Couto et al., 2019). In both cases, the corrected number of haploid seeds for the $i^{th}\;$ inducer, in the $j^{th}$ planting date, at the $k^{th}$ harvested ear, was modeled as $Y_{ijk}∼\;Binomial\;\left(m_{ijk},\;\pi_{ijk}\right)$ , where $Y_{ijk}$ , $m_{ijk}$ , and $\pi_{ijk}$ are the number of haploid seeds, the total number of seeds, and the probability of successful production of haploid seeds, respectively.

The first strategy consisted of fitting a generalized linear model (GLM) with a *logit* link function, expressed:


(Model 1) $$logit\;\left(\pi_{ijk}\right)=log\left(\frac{\pi_{ijk}}{1\;-\;\pi_{ijk}}\right)=\mu +\beta_{j}\;+\tau_{i}$$

Where μ is the intercept, $\beta_{j}$ is the fixed effect of the $j^{th}$ planting date, and $\tau_{i}$ is the fixed effect of the $i^{th}\;$ inducer. The dispersion parameter ϕ  from Model 1 was estimated by:


$$\phi \;=\;\frac{\sum_{k\;=\;1}^{n}r_{k}^{2}}{n-p}$$

Where $r_{k}$ is the Pearson residual of the $k^{th}$ observation, and n-p is the degree of freedom for the deviance residuals. If Pearson’s Chi-Square Statistic suggests a lack of fit from Model 1, then a Quasi-Likelihood inferential approach that accounts for overdispersion was assumed.

The second strategy consisted of fitting a generalized linear mixed model (GLMM) with a *logit* link function, expressed by the following linear predictor:


(Model 2) $$logit\;\left(\pi_{ijk}\right)=log\left(\frac{\pi_{ijk}}{1\;-\;\pi_{ijk}}\right)=\mu +\beta_{j}+\tau_{i}+\epsilon_{ij}$$

Where μ is the intercept, $\beta_{j}$ is the random effect of the $j^{th}$ planting date with $\beta_{j}\;∼N\left(0,\;\sigma_{\beta }^{2}\right)$ , $\tau_{i}$ is the fixed effect of the $i^{th}\;$ inducer, and $\epsilon_{ij}$ is the random effect of the plot with $\epsilon_{ij}\;∼N\left(0,\;\sigma_{\epsilon }^{2}\right)$ . The following two models account for the random effects of individual observations ( $\omega_{k\left(ij\right)}$ , i.e., harvested ears) and both individual observations and plot variation, respectively, as follows:


(Model 3) $$logit\;\left(\pi_{ijk}\right)=log\left(\frac{\pi_{ijk}}{1\;-\;\pi_{ijk}}\right)=\mu +\beta_{j}+\tau_{i}+\omega_{k\left(ij\right)}$$

(Model 4) $$logit\;\left(\pi_{ijk}\right)=log\left(\frac{\pi_{ijk}}{1\;-\;\pi_{ijk}}\right)=\mu +\beta_{j}+\tau_{i}+\epsilon_{ij}+\omega_{k\left(ij\right)}$$

Where $\omega_{k\left(ij\right)\;}∼\;N\left(0,\;\sigma_{\omega }^{2}\right)$ . All the other model terms were previously defined.

Three criteria were used to select the best-fit model: (a) the goodness-of-fit via the Akaike Information Criterion (AIC; Akaike, 1974), (b) the Bayesian Information Criterion (BIC; Schwarz, 1978); and (c) visual inspection using half-normal plots with a simulated envelope (Demétrio et al., 2014) using the R package *hnp* (Moral et al., 2017). Plots of the Pearson residuals were also compared between models. Maximum likelihood estimates for the fixed effects of inducers $\left({\hat{Y}}_{i..}\right)$ from the selected model were further used for GWAS analysis. Asymptotic confidence intervals were obtained using the package *emmeans* (Lenth, 2020). All statistical analyzes were carried out using the *lme4* package (Bates et al., 2015) in the R environment (R Core Team, 2022).

### Genotyping and quality control

Leaf samples were collected when seedlings were at the V3 stage. After lyophilization, they were sent to the International Maize and Wheat Improvement Center (CIMMYT), where they were genotyped with the Diversity Arrays Technology (DArTSeq) platform (Kilian et al., 2012). SNP calling was performed using the DArTsoft analytical pipepeline (https://www.diversityarrays.com) and the version 4 of B73’s reference genome (AGPv4) as a reference. A total of 32,929 SNP markers were initially identified, and 9,007 remained after filtering for a call rate of at least 70% and minor allele frequency (MAF) of 1% (https://dr.lib.iastate.edu/home). The software Beagle 5.0 (Browning et al., 2018) was subsequently used for the imputation of missing data.

### Genome-wide association analyses

We performed marker-trait association using quantitative and binary response variables. The quantitative GWAS was implemented in GAPIT (Lipka et al., 2012) with the multiple loci models (i) BLINK (Huang et al., 2019), (ii) FarmCPU (Liu et al., 2016), and (iii) MLMM (Segura et al., 2012). The estimated HIR from the best-fit phenotypic model was normalized (i.e., transformed) using the *bestNormalize* package in R (Peterson, 2021). The binary GWAS was a case-control and included the 159 inducers considered “cases”, and the panel of 793 noninducers considered “controls” (**Table S1**). In total, 952 genotypes were included. The phenotypes, a binary random variable, were coded as ones for the cases and zeros for the controls, and a mixed logistic regression model was implemented in the R package *milorGWAS* (Milet et al., 2020). The reader is referred to the cited references for details on these GWAS models.

**Figure 1:**
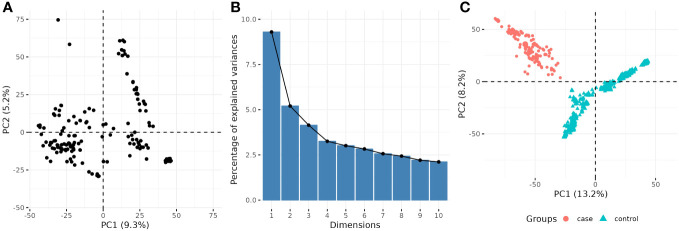
Scatterplot of two first principal components (PCs) from principal component analysis (PCA) of SNP matrix for **(A)** quantitative and **(C)** binary GWAS. The percentage of explained variance for each PC in the quantitative GWAS is shown in **(B)**.

For the quantitative GWAS, population/family structure was accounted by the additive genomic relationship G (VanRaden, 2008), and the first seven principal components (PCs) obtained from principal component analysis (PCA, built-in R function *prcomp*) of the numerical matrix of SNP markers, calculated via singular value decomposition (**Figures 1A, B**). For the cc-GWAS, the cases and controls grouping of genotypes were completely confounded by the first two PCs (**Figure 1C**), and hence population structure was only modeled by the matrix G. It is unlikely such correction can fully adjust for the population structure; hence, a permutation analysis was performed as follow: the phenotypes (zeros and ones) were shuffled under the null hypothesis of no association between markers and phenotypes, and the lowest *p-value* recorded. This process was repeated 10,000 times, and the 95% quantile of the distribution of the *-log10(p-values)* was used as a reference threshold. In addition to the permutation analysis for the cc-GWAS, SNPs were declared statistically significant for both GWAS analyses at an FDR-adjusted *p-value* of less than 0.05.

Lastly, we calculated the genome-wide (i.e., across chromosomes) pairwise linkage disequilibrium (LD) using the function *LD.decay* from the *sommer* package (Covarrubias-Pazaran, 2016). The LD decay was determined by locally-weighted polynomial regression (LOESS, build-in R function) with the physical distance between markers as a function of the coefficient of determination $\left(r^{2}\right)$ . The threshold $r^{2}$ value when estimating LD decay was calculated with the $95^{th}$ percentile of the LD distribution between unlinked markers [*LD.decay(…, unlinked = TRUE, gamma = 0.95)*]. The search browser in MaizeGDB (https://www.maizegdb.org/gbrowse) was used to identify the putative candidate genes in the interval of significant SNPs. Maize B73 RefGen_v4 version was used to locate the candidate genes.

## Results

### Phenotypic analyses

**Figure 2:**
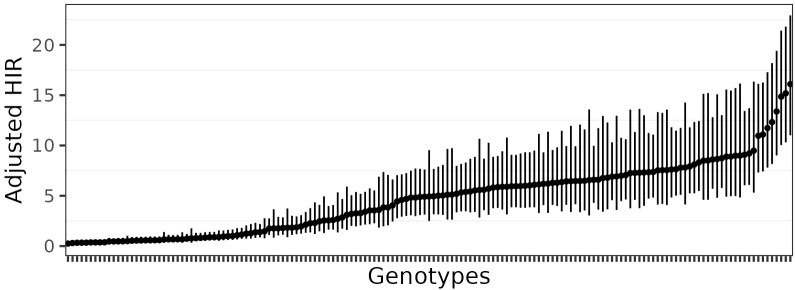
Estimated HIR with asymptotic confidence intervals estimated from Model 4.

**Figure 3:**
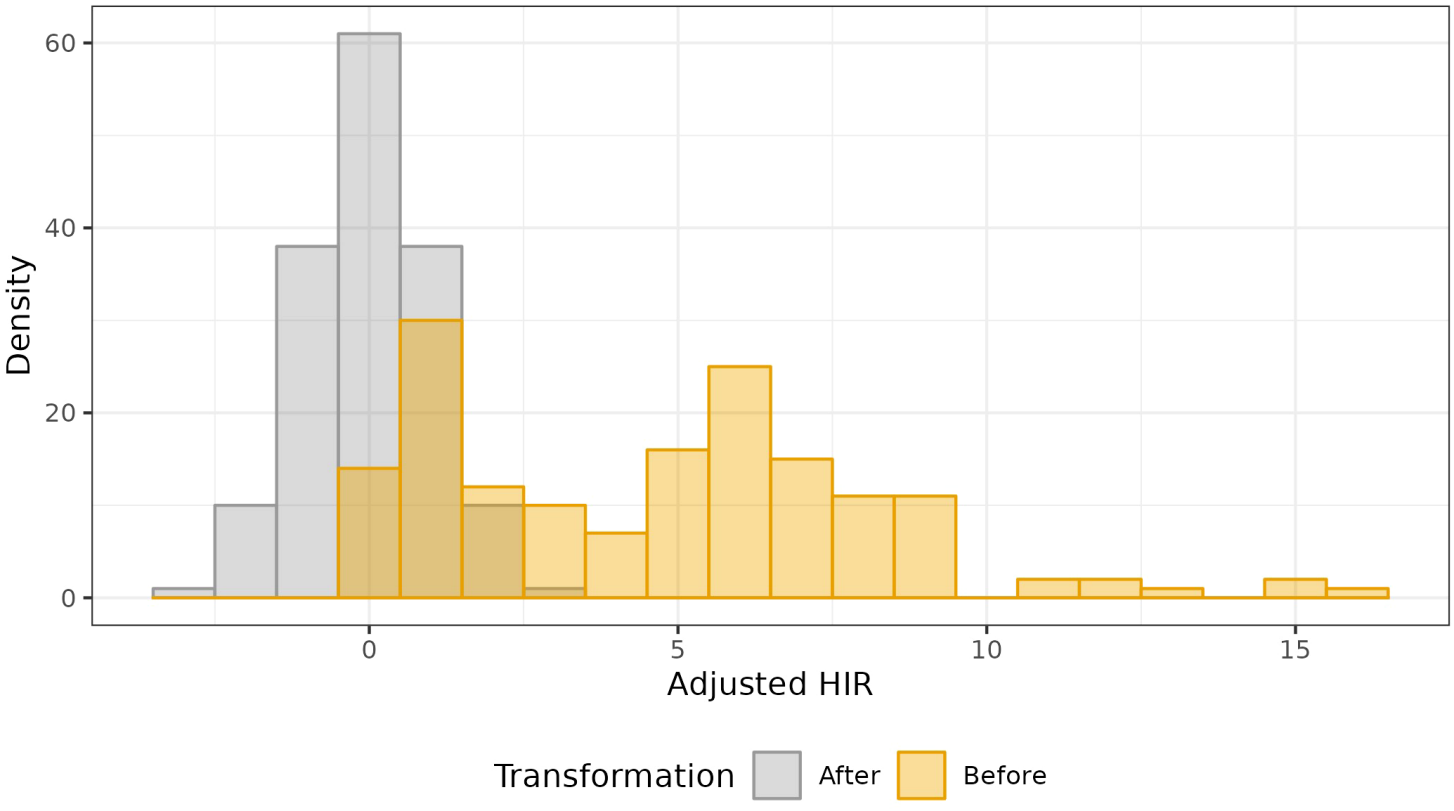
Data transformation of the estimated HIR from Model 4 to perform the quantitative GWAS.

Estimates of HIR with asymptotic confidence intervals were obtained with Model 4 (**Figure 2**; **Table S2**). For the selection of the best-fit phenotypic model, the AIC values ranged from 14702.24 (Model 4) to 16932.52 (Model 1), and for the BIC criterion, from 15668.51 (Model 4) to 17886.93 (Model 1). In addition, Model 1 was adjusted with the Quasi-likelihood inference due to overdispersion (**Table 1**). Thus, both selection criteria suggested that Model 4, which included random effects for planting dates, plots, and individual observations (i.e., harvested ears), was the best-fit model for the observed data. The visual inspection of the half-normal and Pearson residuals plots also showed the superior fit of Model 4 (**Figures S1** and **S2**). Prior to GWAS analyses, the estimated HIR values were transformed to ensure normality (**Figure 3**).

**Table 1:** Goodness-of-fit for Models 1 to 4. Model 4 is the best-fit model.

Model	Log-likelihood	AIC	BIC	Variance components			Dispersion parameter*
				Block	Plot	Ears	
1	-8305.26	16932.52	17886.93	–	–	–	3.05
2	-7889.48	16102.95	17063.30	0.01	0.10	–	–
3	-7257.02	14838.03	15798.38	0.01	–	0.26	–
4	-7188.12	14702.24	15668.51	0.01	0.08	0.20	–

*Model fitted with Quasi-likelihood approach, "-" symbol indicates no data.

### Genome-wide association analyses

**Figure 4:**
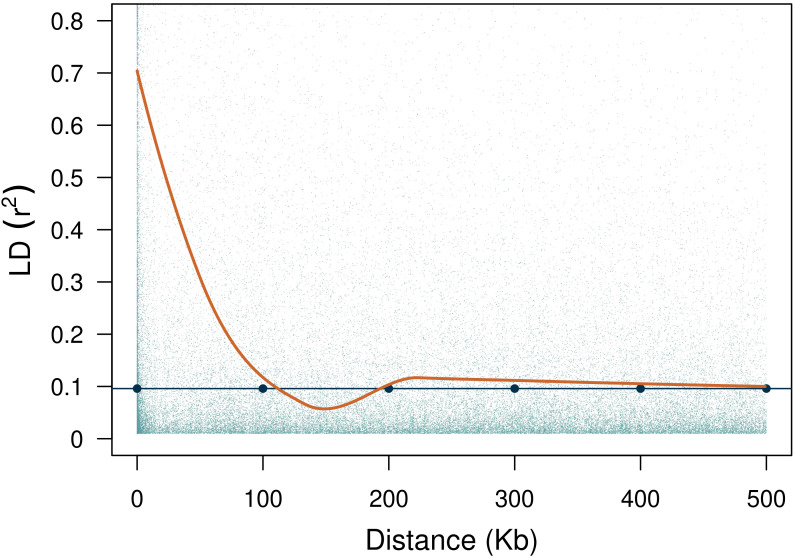
LD decay with an estimated threshold value of 0.096. The fitted LOESS curve is shown in orange.

**Figure 5:**
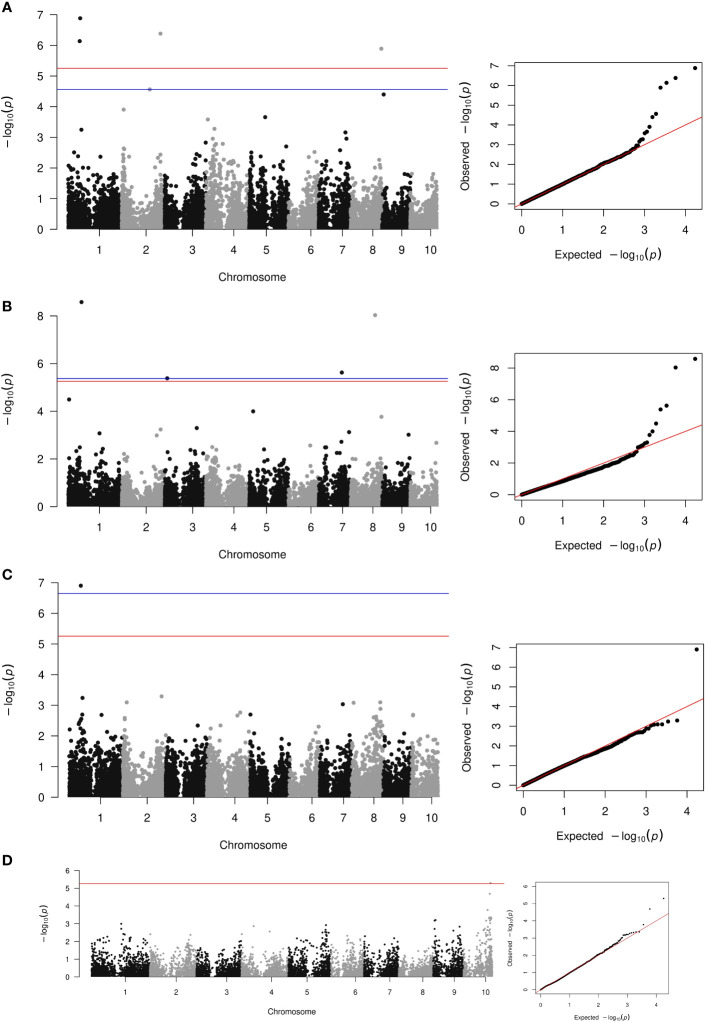
Manhattan and qq-plot of the quantitative GWAS using Blink **(A)**, FarmCPU **(B)**, MLMM **(C)**, and the case-control GWAS **(D)**.

**Figure 6:**
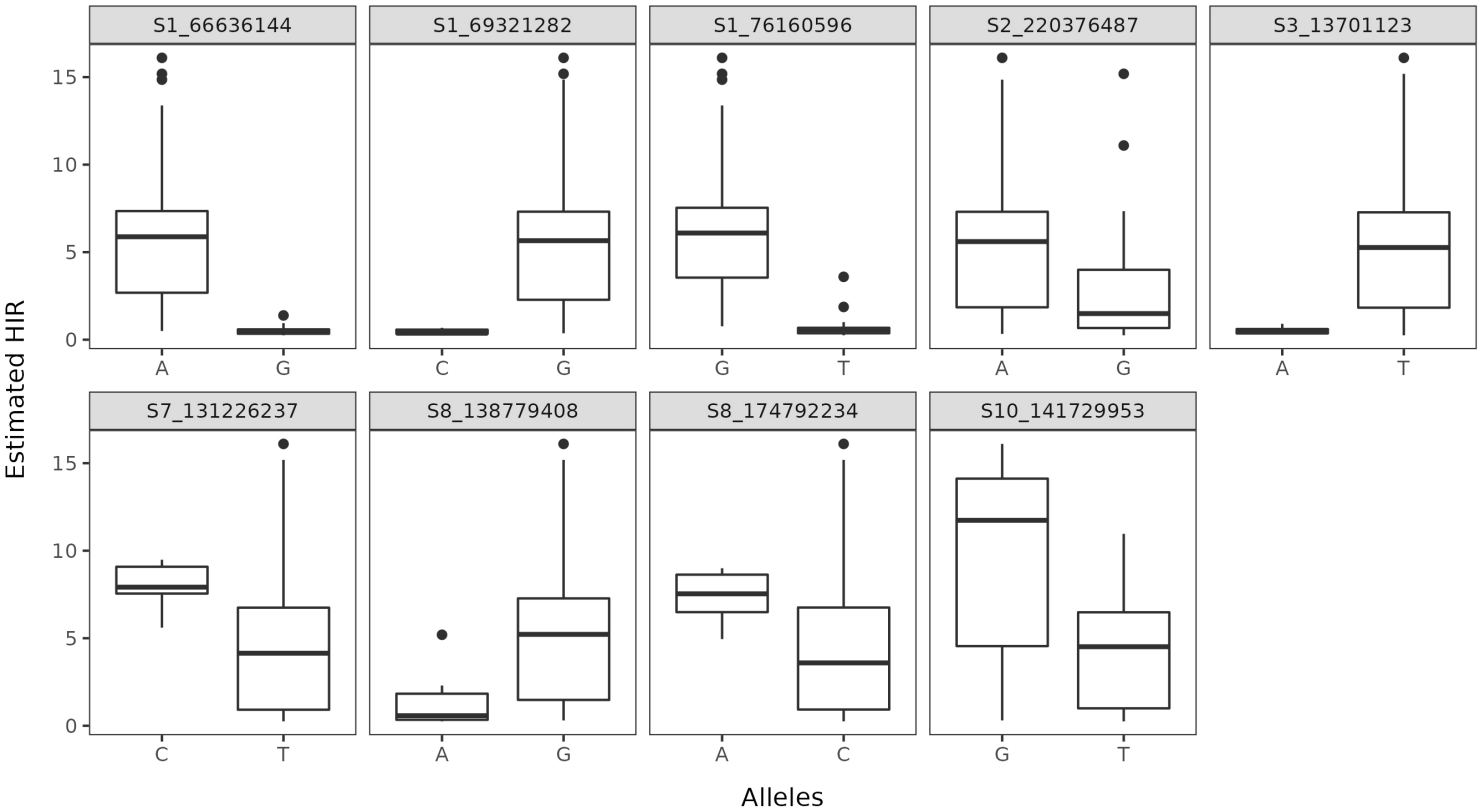
Allelic distribution as a function of the estimated HIR at each statistically significant SNP.

The average rate of LD decay at the $r^{2}$ estimated threshold of 0.096 was of 113 Kb (**Figure 4**). The reference threshold obtained from the permutation analysis for the cc-GWAS was of 4.89, whereas the *-log10(p-value)* of the statistically significant SNP marker on chromosome 10 was of 5.30, with an adjusted FDR value of 0.04 (**Figure 5D**). In the quantitative GWAS, significant SNPs were detected on chromosomes 1, 2, 3, 7 and 8 (**Figures 5A–C**). The significant SNPs detected in chromosome 1 were located in positions 66.6 Mb (S1_66636144), 69.3 Mb (S1_69321282) and 76.1 Mb (S1_76160596), flaking the region of *MTL/ZmPLA1/NLD*, which is located in position 69.4 Mb. The significant SNP detected on chromosome 2 (S2_220376487) is located at position 220.3 Mb and does not overlap with the QTL detected by Deimling et al. (1997) on the same chromosome. The significant SNP detected on chromosome 3 (S3_13701123) is located at position 13.7 Mb and does not overlap with any of chromosome 3 QTL’s previously identified by Prigge et al. (2012b). The significant SNP detected on chromosome 7 (S7_131226237) is located at position 131.2 Mb, and also does not overlap with the chromosome 7 QTL previously identified by Prigge et al. (2012b). The two significant SNP detected on chromosome 8 (S8_138779408 and S8_174792234) are located at positions 138.7 and 174.7 Mb, respectively. There are no reports of HIR QTL on this chromosome in the literature. The cc-GWAS detected a significant SNP (S10_141729953) at position 141.7 Mb on chromosome 10. To the best of our knowledge, there are no reports of HIR QTL on chromosome 10 in the literature. A summary overview of the statistically significant SNPs is given in **Table 2**, and the allelic distribution as a function of the estimated HIR in **Figure 6**. For the binary GWAS, a classification table differentiating the number of cases and controls according to the genotyped allele is presented (**Table 3**).

**Table 2:** Summary of the statistically significant SNPs containing name, chromosome, position in base pairs, alleles, minor allele frequency (MAF), and models’ name. C-C stands for cc-GWAS.

SNP marker	Chromosome	Position	Alleles	MAF^a^	BLINK	FarmCPU	MLMM	C-C
S1_66636144	1	66636144	A/G	0.21	x		x	
S1_69321282	1	69321282	C/G	0.13	x			
S1_76160596	1	76160596	G/T	0.22		x		
S2_220376487	2	220376487	A/G	0.18	x			
S3_13701123	3	13701123	A/T	0.08		x		
S7_131226237	7	31226237	C/T	0.03		x		
S8_138779408	8	138779408	A/G	0.08		x		
S8_174792234	8	74792234	A/C	0.06	x			
S10_141729953	10	141729953	G/T	0.13				x

a MAF was calculated respective to the population used (i.e., 159 or 952 genotypes).

**Table 3:** Counts of number of genotypes for SNP marker S10_141729953 from cc-GWAS analysis.

Allele	Cases	Controls
G	11	789
T	146	3

### Identification and annotation of candidate genes

The physical locations of the nine significant SNPs identified were recorded using the B73 RefGen_v4 based on the LD decay distance. The candidate genes were identified by the genome browser search available at Maizegdb (https://www.maizegdb.org/gbrowse). A total of 41 candidate genes with descriptions were found, out of which, 37 were protein-coding, and the remaining 4 were not annotated. The chromosomal distribution of these candidate genes included 12 located on chromosome 1, 8 on chromosome 2, 5 on chromosome 3, 5 on chromosome 7, 3 on chromosome 8, and 8 on chromosome 10. The functional domain of each of the candidate genes is provided in **Table S3**.

## Discussion

The widespread use of the DH technique in maize was influenced by the increase of HIRs to manageable levels and the addition of anthocyanin-based phenotypic markers in maternal inducers. These two fields have recently seen remarkable advancements. For instance, Chen et al. (2022) recently developed an efficient and accurate (99.1% accuracy) haploid selection marker through co-expression of two transcription factor genes (*ZmC1* and *ZmR2*) in novel haploid inducer line called ‘Maize Anthocyanin Gene InduCer 1 (MAGIC1)’. Shortly after, Wang et al. (2023) showed that expression of *RUBY* enabled 100% accuracy of haploid identification based on deep betalain pigmentation. The development of a group of inducers with numerous anthocyanin markers and HIRs above 12.0% allowed for effective haploid generation and screening (Rotarenco et al., 2010). The identification of the *MTL/ZmPLA1/NLD* (Gilles et al., 2017b; Kelliher et al., 2017; Liu et al., 2017) DUF679 (Zhong et al., 2019), *ZmPLD3* (Li et al., 2021) and *ZmPOD65* (Jiang et al., 2022) genes contributed to a better understanding of the molecular mechanisms of haploid induction (Jacquier et al., 2020). Previous research (Lashermes and Beckert, 1988; Deimling et al., 1997; Röber, 1999; Prigge et al., 2012b; Liu et al., 2015) revealed evidence for the polygenic regulation of HIR and identified various QTL controlling this trait. To increase the effectiveness of inducer breeding and, as a result, lower the cost of DH line production, it is critical to identify and validate the influence of QTL affecting HIR. To achieve this goal, we designed and performed a GWAS with a large and diverse panel of inducers. In a previous GWAS for HIR, the induction ability of 53 inducers was evaluated in distinct donors (Hu et al., 2016). Comparing the induction ability obtained through crosses with different donors is not ideal, since inducibility and HIR effects might be confounded (Randolph, 1940; Chase, 1952; Lashermes and Beckert, 1988; Eder and Chalyk, 2002; De La Fuente et al., 2018). In our study, 159 inducers were crossed to the same donor at two different planting dates.

### Genome-wide association analyses

To this date, just a few studies were performed to identify QTL affecting the HIR of maize maternal haploid inducers (Deimling et al., 1997; Barret et al., 2008; Prigge et al., 2012b; Hu et al., 2016). However, multiple studies have been performed to fine-map or validate the function of previously identified QTL (Dong et al., 2013; Liu et al., 2015; Gilles et al., 2017b; Kelliher et al., 2017; Liu et al., 2017; Nair et al., 2017; Zhong et al., 2019; Li et al., 2021).

In this study, we used four different GWAS methods, including BLINK, FarmCPU, MLMM, and cc-GWAS, to identify potential associations with single nucleotide polymorphisms (SNPs). We identified a total of nine SNPs that were significantly associated with the trait. They correspond to different chromosomal origins, validating the polygenic control of HIR in maize, as previously reported (Lashermes and Beckert, 1988; Deimling et al., 1997; Röber, 1999; Prigge et al., 2012b; Liu et al., 2015). For instance, two SNPs (S8_138779408 and S8_174792234) were identified on chromosome-8 and a SNP (S10_14172995) chromosome-10. No QTLs were previously reported on these chromosomes. However, some of the significant SNPs that we identified are closely linked to the previously discovered QTLs for HIR. The marker S1_69321282, at position 69.3 Mb on chromosome 1, is closely linked to the *MTL/ZmPLA1/NLD* gene at position 69.4 Mb (identified in the region *qhir11*). Similarly, marker S1_76160596 (position 76.1 Mb), is located inside the *qhir12* region which Hu et al. (2016) found a higher CHE score for *qhir12* than for *qhir11*. Interestingly, Hu et al. (2016) also reported that the *qhir12* haplotype was exclusively found in inducers, whereas the *qhir11* haplotype could be found in 2.7% of the non-inducers. Nair et al. (2017) showed the significance of *qhir11* region compared *qhir12* for inducing haploid embryo in maize. At the same time, it was discovered that *qhir11* harbours the *MTL/ZmPLA1/NLD* allele that possesses major effect on haploid induction in maize. The differences in germplasm, genotyping method, statistical models and sample size might explain the divergence between earlier studies and our results. The inbred panel used in this study essentially carried the mutant *MTL/ZmPLA1/NLD* allele and therefore it is obvious that the gene would not be detected in our quantitative GWAS.

We also found two significant markers, S2_220376487 at 220.3 Mb and S7_13226237 at 132.2 Mb on chromosome 2 and chromosome 7, respectively, in regions that does not overlap QTL identified on chromosome 2 (Deimling et al., 1997) and chromosome 7 (Prigge et al., 2012b). Although on the same chromosome, we report these new genomic regions that could influence haploid induction. PVE (phenotypic variance explained) is an important metric used in GWAS to quantify the contribution of SNP for trait value and indicator of the biological relevance of the SNP. PVE for the significant SNPs varied from 5.9% to 37% explaining substantial variance for HIR detected in our study. SNPs S1_66636144, located at position 66.6 Mb on chromosome 1 (linked to *MTL/ZmPLA1/NLD* gene), S8_174792234, located at position 174.7 Mb on chromosome 8 and S7_131226237, located at position 131.2 Mb on chromosome 7, with higher PVE values of 37%, 25% and 21%, respectively, are likely to be more important for the trait and are more likely to have a direct impact on the HIR. Notably, detection of S1_66636144 at position 66.6 Mb on chromosome 1 by BLINK and MLMM models makes it as potential region for investigation, in addition to SNP S8_174792234, at position 174.7 Mb on chromosome 8 which is reported first time for the HIR. A smaller p-value suggests that the association is less likely to be due to chance and more likely to be real. Apart from S1_66636144 at 66.6 MB on chromosome 1, the marker S8_174792234 at position 174.7 Mb on chromosome 8 possessed the lowest p-value followed by S1_69321282 on chromosome 1 at 69.3 Mb (tightly linked to *MTL/ZmPLA1/NLD* gene) compared to the two other markers (S1_66636144 and S1_76160596 at 66.6 and 76.1 Mb, respectively) detected on chromosome 1. Given their high significance, it is interesting that they have not been detected in other mapping studies (Deimling et al., 1997; Barret et al., 2008; Prigge et al., 2012b; Hu et al., 2016). This failure of detection might have been caused by the absence of genetic variation at these QTL in the germplasm used in other studies.

In a cc-GWAS, we compared inducers (cases) to noninducers (controls) to identify genetic variants that are associated with the haploid induction. As a result, we identified a significant novel genetic region, close to marker S10_141729953, which is located at position 141.7 Mb of chromosome 10. Therefore, the identification of S10_141729953 as associated with HIR can help improve our understanding of the genetic factors involved in this trait. The failure of detection of this region might have been due to the absence of genetic variation at these QTL in the germplasm used in other studies (Lashermes and Beckert, 1988; Deimling et al., 1997; Röber, 1999; Prigge et al., 2012b; Liu et al., 2015). For marker S10_141729953, this seems to be a plausible explanation since the G allele was almost exclusively found in MHI and in its progeny. Another explanation might be that their effect is dependent on the presence of the *mtl/zmpla1/nld* allele, similarly to what is described for the *zmdmp* allele. This also seems to be the case for inducers carrying the G allele at marker S10_141729953. From those, the only two which do not have the common inducer haplotype at the *MTL/ZmPLA1/NLD* region (HUT45 and HUT71), display an HIR< 1.0% at all planting dates. The population structure for the cc-GWAS analysis was only controlled with the additive genomic relationship matrix due to the confounding between the cases and controls grouping of genotypes with the first two principal components. Thus, further research is needed to ensure that marker S10_141729953 was not a spurious association.

### Candidate genes

We searched the putative candidate genes in the interval of 56kb on both sides of nine significant markers identified in the study. The aldose 1-epimerase enzyme-coding gene Zm00001d029341 has been shown to be expressed in meiotic tassel (Stelpflug et al., 2016). This could be one of the candidate genes for inducing haploid in maize. The gene Zm00001d029558, which encodes for a pathogenesis-related protein 1, contains the marker S1_76160596. Pollen allergen properties of pathogenesis-related proteins have been reported in a variety of species (Huang et al., 1997 and Huang et al., 2016). However, there was no evidence of Zm00001d029558 expression in pollen (Walley et al., 2016). S1_76160596, the same marker, is found 19.9 kb downstream of the gene Zm00001d029559, which encodes an EID1-like F-box protein 2. EID1 (Empfindlicher im Dunkelroten Licht 1) is a F-box protein that belongs to the SKP1-Cullin1-F-box (SCF) proteomic complexes that target protein degradation (Marrocco et al., 2006). F-box-like protein 17 (FBL17) in Arabidopsis targets the degradation of two CDKA;1 inhibitors, KRP6 and KRP7 (Kim et al., 2008). Arabidopsis fbl17 mutants occasionally fail to divide and produce pollen grains with a single sperm cell, resulting in single fertilization events (Kim et al., 2008). A-type cyclin-dependent kinase 1 (CDKA;1) is a homolog of the human serine/threonine protein kinase cdc2. Arabidopsis CDKA;1 mutation delay pollen generative cell division (Aw et al., 2010), resulting in the delivery of a single sperm cell to the ovary. It is possible that gene Zm00001d029559 interferes with maize’s CDKA;1 via the same pathway described in Arabidopsis, influencing HIR by increasing the frequency of single fertilization events, which is one of two processes thought to cause haploid induction.

The marker S10_141729953 is in the intragenic region of gene Zm00001d026242, which was reported to be either exclusively or highly expressed in pollen grains (Davidson et al., 2011; Walley et al., 2016). Zm00001d026242 currently does not have an annotation, but the National Center for Biotechnology Information (NCBI) nucleotide Basic Local Alignment Search Tool (BLAST) shows that its nucleotide sequence is 88.9% and 83.0% identical to the *Sorghum bicolor*’s and maize’s *kokopelli* (*KPL*) mRNAs, respectively. *Kokopelli* was first described in *Arabidopsis thaliana*, where it was named after the Native American deity of male fertility (Ron et al., 2010). In maize, *kokopelli* is located on chromosome 2 (NCBI’s sequence ID: XP_008671112.1), whereas in sorghum it is located on chromosome 6 (NCBI’s sequence ID: XP_021319809.1). *Kokopelli* encodes for a natural cis-antisense siRNA (cis-nat-siRNA), which is a class of small regulatory RNAs found in various eukaryotic species. Even though two apparently normal sperms are delivered to embryos sacs by the pollen tubes of the Arabidopsis *kpl* mutant, single fertilization was observed in approximately 40% of the ovules fertilized (Ron et al., 2010). In Arabidopsis, *KPL* forms a sperm-specific nat-siRNA pair with the inversely transcribed gene *ARIADNE14* (*ARI14*), which encodes for a putative ubiquitin E3 ligase, and whose overexpression decreases seed set (Ron et al., 2010). Protein degradation occurs during gametogenesis in plants and animals and is required for successful fertilization (Dickins et al., 2002; Suzumori et al., 2003; Roest et al., 2004; Ron et al., 2010; Wang et al., 2013). Altogether, these results suggest a possible involvement of Zm00001d026242 in fertilization and haploid induction. Recently, Jacquier et al. (2023) validated the role of *Atkpl* (KOKOPELLI, AT5G63720) mutant for triggering *in planta* maternal haploid induction.

### Applicability of the findings to inducer breeding

The detection of candidate genes in six maize chromosomes is in agreement with other studies that suggested a polygenic control of HIR (Lashermes and Beckert, 1988; Deimling et al., 1997; Röber, 1999; Prigge et al., 2012b; Liu et al., 2015; Nair et al., 2017). The presence of genes with strong effects, like *MTL/ZmPLA1/NLD*, *ZmDMP*, *ZmPLD3*, *ZmPOD65* and the QTL identified in this study on chromosomes 1, 8 and 10, suggest that some form of MAS, such as F_2_-enrichment or marker-assisted backcrossing, might be efficient breeding strategies for inducer development. Employing genomic selection or predicted cross value (Han et al., 2017) during or after the fixation of these major genes, can help to capture the best combination of small effect QTL (Almeida et al., 2020). Phenotypic selection for HIR is very laborious and time-consuming, since multiple cross-pollinations need to be performed to obtain reliable estimates of HIR. Moreover, visual discrimination of haploid and diploid seeds is time-consuming and error-prone, since *R1-nj* expression is affected by environmental conditions, seed morphology and anthocyanin inhibitor genes present in the donor background (Burr et al., 1996; Della Vedova et al., 2005; Röber et al., 2005; Kebede et al., 2011; Prigge et al., 2011; Prigge et al., 2012a; Prigge et al., 2012b).

The development of high oil content (OC) inducers allows automated haploid selection, which greatly facilitates accurate estimation of HIRs. However, OC is a quantitatively inherited character (Moreno-Gonzalez et al., 1975; Berke and Rocheford, 1995; Laurie et al., 2004; Môro et al., 2012), as are other traits important to inducers, such as plant height, tassel size and disease tolerance (Gallavotti et al., 2004; Satoh-Nagasawa et al., 2006; Peiffer et al., 2014; Tembo et al., 2014; Zhang et al., 2016; Almeida et al., 2020). Employing genomic selection to simultaneously improve multiple polygenic traits using optimization criteria (Akdemir et al., 2019) dilutes the costs of genotyping and becomes more cost-effective as the number of traits that otherwise would have to be phenotyped increase. Nevertheless, visual discrimination of haploid seeds through the *R1-nj* anthocyanin marker is still performed in the majority of medium to small scale breeding programs employing the DH technique. Therefore, the economic benefits of using inducers with high HIR is still important.

## Data availability statement

The original contributions presented in the study are included in the article/**Supplementary Material**. The genetic polymorphism data is available at https://doi.org/10.25380/iastate.24185784.v2. Further inquiries can be directed to the corresponding author.

## Author contributions

HT, UF, and TL designed this project; HT, UF, VR, and TL developed the inducer panel used in this study; HT, VA, and EP performed most of the HIR phenotypic selection; MK and WB analyzed the phenotypic and genomic data; HT and RZ executed the candidate gene analysis; HT, MK, RZ and TL wrote this manuscript; TL supervised the research work; All authors contributed to the article and approved the submitted version.

## Supplementary material

Supplementary Figure 1 Half-normal plots with a simulated envelope for **(A)** Model 1, **(B)** Model 1 with Quasi-likelihood inference, **(C)** Model 2, **(D)** Model 3, and **(E)** Model 4.

Supplementary Figure 2 Index Pearson residual plots for **(A)** Model 1, **(B)** Model 2, **(C)** Model 3, and **(D)** Model 4.
