# Supplementary figures and images for: Genetic basis of maize maternal haploid induction beyond MATRILINEAL and ZmDMP
Source: Front Plant Sci. 2023 Oct 4;14:1218042. doi: 10.3389/fpls.2023.1218042 (PMC10582762; doi:10.3389/fpls.2023.1218042)

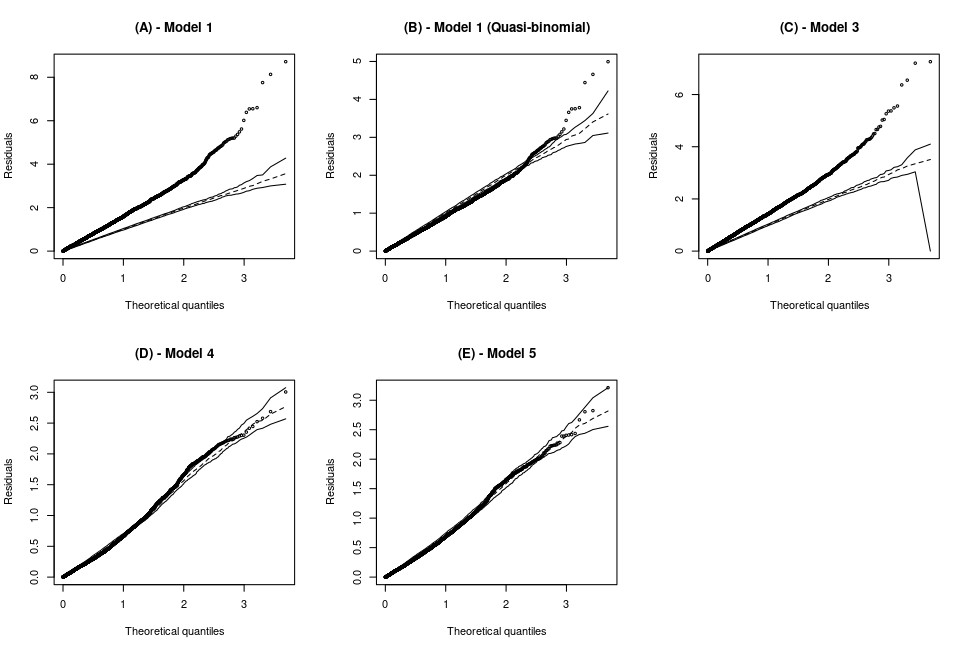

Supplement: Supplementary Figure 1 — Half-normal plots with a simulated envelope for (A) Model 1, (B) Model 1 with Quasi-likelihood inference, (C) Model 2, (D) Model 3, and (E) Model 4. [file Image_1.jpeg]

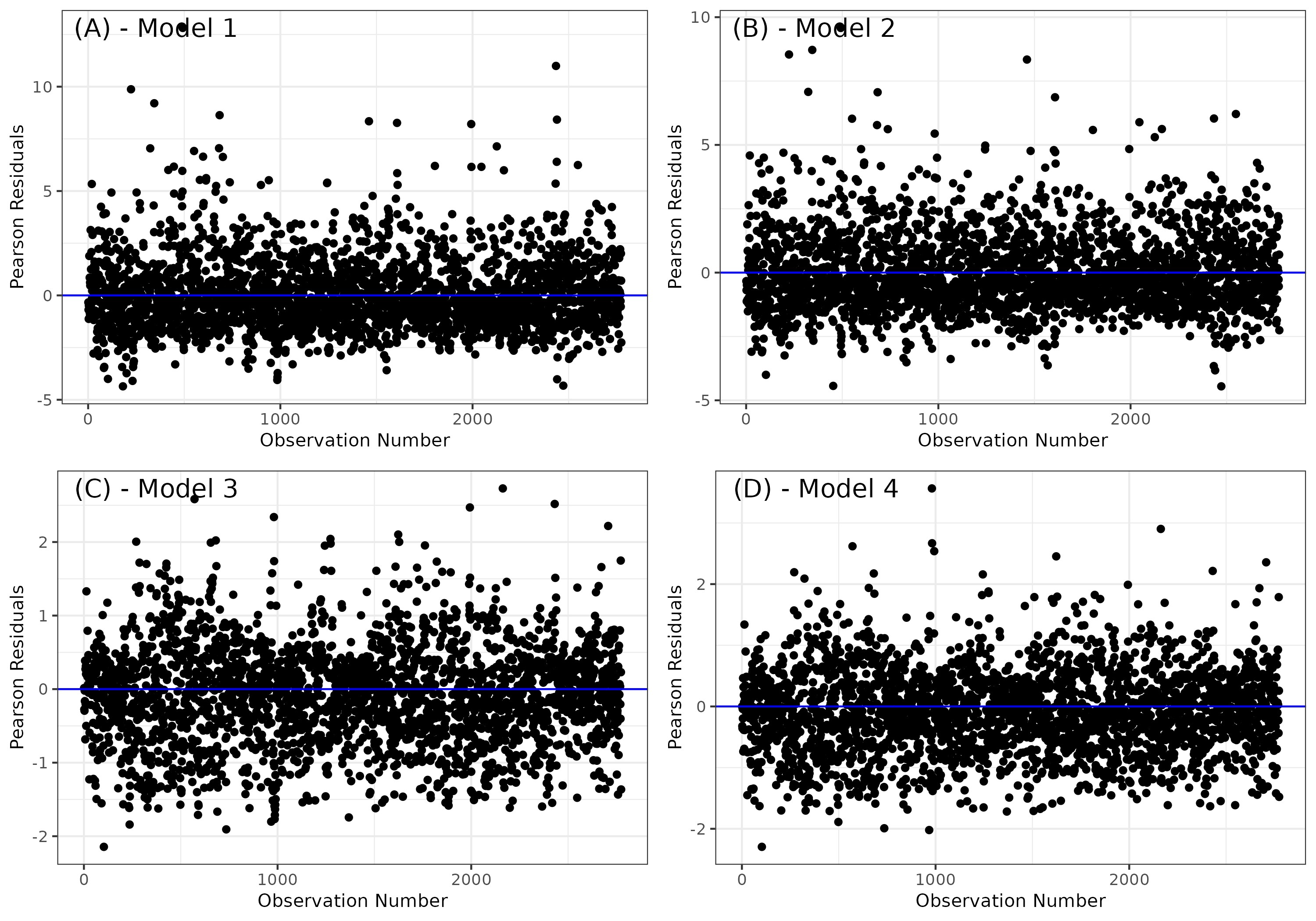

Supplement: Supplementary Figure 2 — Index Pearson residual plots for (A) Model 1, (B) Model 2, (C) Model 3, and (D) Model 4. [file Image_2.jpeg]
