# Supplementary material for: Genetic basis of maize maternal haploid induction beyond MATRILINEAL and ZmDMP
Source: Front Plant Sci. 2023 Oct 4;14:1218042. doi: 10.3389/fpls.2023.1218042 (PMC10582762; doi:10.3389/fpls.2023.1218042)
Supplement: Supplementary file 5 [file Table_3.docx]

**Table S3: Information of the most promising candidate genes identified in this study**

| **S. No.** | **Gene Stable ID** | **Chr: Start-End (bp)** | **Functional domain** |
| --- | --- | --- | --- |
|  | Zm00001d029339 | Chr1: 66,568,393-66,574,338 | bHLH-transcription factor |
|  | Zm00001d029340 | Chr1: 66,592,542-66,594,846 | rRNA binding |
|  | Zm00001d029341 | Chr1: 66,635,784-66,637,299 | aldose 1-epimerase activity |
|  | Zm00001d029342 | Chr1: 66,637,359-66,648,456 | tRNA pseudouridine synthase |
|  | Zm00001d029343 | Chr1: 66,648,726-66,655,094 | actin filament binding |
|  | Zm00001d029408 | Chr1: 69,295,860-69,299,198 | ARF-related protein |
|  | Zm00001d029409 | Chr1: 69,302,141-69,303,331 | Chaperone DnaJ-domain superfamily protein |
|  | Zm00001d029410 | Chr1: 69,320,905-69,324,756 | Carboxypeptidase |
|  | Zm00001d029558 | Chr1: 76,160,502-76,161,041 | pathogenesis-related protein14 |
|  | Zm00001d029559 | Chr1: 76,180,529-76,181,281 | EID1-like F-box protein 2 |
|  | Zm00001d029560 | Chr1: 76,209,755-76,212,824 | RING/U-box superfamily protein |
|  | Zm00001d029561 | Chr1: 76,213,706-76,225,802 | Putative ubiquitin conjugation factor E4 |
|  | Zm00001d007021 | Chr2: 220,334,883-220,340,521 | Unknown |
|  | Zm00001d007022 | Chr2: 220,339,404-220,345,439 | Squamosa promoter-binding-like protein 14 |
|  | Zm00001d007023 | Chr2: 220,347,263-220,352,474 | ATP-dependent DNA helicase |
|  | Zm00001d007024 | Chr2: 220,363,460-220,365,167 | Pentatricopeptide repeat-containing protein |
|  | Zm00001d007025 | Chr2: 220,365,744-220,375,203 | Unknown |
|  | Zm00001d007026 | Chr2: 220,375,885-220,377,718 | Exonuclease domain-containing protein |
|  | Zm00001d007027 | Chr2: 220,378,885-220,379,460 | Ubiquitin-protein ligase CIP8 |
|  | Zm00001d007029 | Chr2: 220,383,064-220,388,898 | CDPK-related kinase 1 |
|  | Zm00001d039745 | Chr3: 13,650,821-13,654,113 | Protein phosphatase |
|  | Zm00001d039746 | Chr3: 13,655,404-13,661,672 | ATP-dependent RNA helicase |
|  | Zm00001d039747 | Chr3: 13,687,680-13,693,484 | Transcription initiation factor TFIID subunit |
|  | Zm00001d039748 | Chr3: 13,694,033-13,694,665 | Pollen Ole e 1 allergen and extension family |
|  | Zm00001d039749 | Chr3: 13,697,952-13,705,516 | Receptor like protein 7 |
|  | Zm00001d020750 | Chr7: 131,175,224-131,180,460 | Carboxypeptidase |
|  | Zm00001d020751 | Chr7: 131,205,805-131,211,307 | Carboxypeptidase |
|  | Zm00001d020752 | Chr7: 131,208,848-131,215,427 | Carboxypeptidase |
|  | Zm00001d020754 | Chr7: 131,220,206-131,228,869 | Carboxypeptidase |
|  | Zm00001d020755 | Chr7: 131,253,170-131,259,045 | Unknown |
|  | Zm00001d012451 | Chr8: 174,779,387-174,785,522 | CTD small phosphatase-like protein |
|  | Zm00001d012452 | Chr8: 174,783,486-174,791,549 | Protein kinase superfamily protein |
|  | Zm00001d012456 | Chr8: 174,832,321-174,839,594 | Jasmonate-regulated gene 21 |
|  | Zm00001d026238 | Chr10: 141,680,042-141,685,861 | Protein TRANSPARENT TESTA 12 |
|  | Zm00001d026239 | Chr10: 141,705,335-141,710,281 | GPCR-type G protein 2 |
|  | Zm00001d026240 | Chr10: 141,714,589-141,718,409 | Growth-regulating factor |
|  | Zm00001d026241 | Chr10: 141,726,352-141,728,004 | EF hand family protein |
|  | Zm00001d026242 | Chr10: 141,728,452-141,731,631 | Unknown |
|  | Zm00001d026243 | Chr10: 141,735,511-141,738,663 | Multiple organellar RNA editing factor 2 chloroplastic |
|  | Zm00001d026244 | Chr10:141,740,263-141,743,415 | Putrescine-binding periplasmic protein-related |
|  | Zm00001d026245 | Chr10: 141,743,929-141,746,414 | Trihelix transcription factor GT-3b |
